# Early activation of quorum sensing in *Pseudomonas aeruginosa *reveals the architecture of a complex regulon

**DOI:** 10.1186/1471-2164-8-287

**Published:** 2007-08-22

**Authors:** Martin Schuster, E Peter Greenberg

**Affiliations:** 1 Department of Microbiology, University of Washington, Box 357242, 1959 NE Pacific St., Seattle, WA 98195, USA; 2 Current address: Department of Microbiology, Oregon State University, 220 Nash Hall, Corvallis, OR 97331, USA

## Abstract

**Background:**

Quorum-sensing regulation of gene expression in *Pseudomonas aeruginosa *is complex. Two interconnected acyl-homoserine lactone (acyl-HSL) signal-receptor pairs, 3-oxo-dodecanoyl-HSL-LasR and butanoyl-HSL-RhlR, regulate more than 300 genes. The induction of most of the genes is delayed during growth of *P. aeruginosa *in complex medium, cannot be advanced by addition of exogenous signal, and requires additional regulatory components. Many of these late genes can be induced by addition of signals early by using specific media conditions. While several factors super-regulate the quorum receptors, others may co-regulate target promoters or may affect expression posttranscriptionally.

**Results:**

To better understand the contributions of super-regulation and co-regulation to quorum-sensing gene expression, and to better understand the general structure of the quorum sensing network, we ectopically expressed the two receptors (in the presence of their cognate signals) and another component that affects quorum sensing, the stationary phase sigma factor RpoS, early in growth. We determined the effect on target gene expression by microarray and real-time PCR analysis. Our results show that many target genes (e.g. *lasB *and *hcnABC*) are directly responsive to receptor protein levels. Most genes (e.g. *lasA*, *lecA*, and *phnAB*), however, are not significantly affected, although at least some of these genes are directly regulated by quorum sensing. The majority of promoters advanced by RhlR appeared to be regulated directly, which allowed us to build a RhlR consensus sequence.

**Conclusion:**

The direct responsiveness of many quorum sensing target genes to receptor protein levels early in growth confirms the role of super-regulation in quorum sensing gene expression. The observation that the induction of most target genes is not affected by signal or receptor protein levels indicates that either target promoters are co-regulated by other transcription factors, or that expression is controlled posttranscriptionally. This architecture permits the integration of multiple signaling pathways resulting in quorum responses that require a "quorum" but are otherwise highly adaptable and receptive to environmental conditions.

## Background

In *Pseudomonas aeruginosa*, two acyl-homoserine lactone quorum sensing (acyl-HSL QS) systems, LasR-LasI and RhlR-RhlI, control the expression of partially overlapping sets of genes. Many of the regulated genes are implicated in virulence and biofilm formation of this opportunistic pathogen. LasI and RhlI are enzymes that synthesize the acyl-HSL signals 3-oxo-dodecanoyl (3OC12)-HSL and butanoyl (C4)-HSL, respectively. LasR and RhlR are receptors that specifically bind the signal generated by their cognate synthases to regulate transcription of target genes [1-5]. Three independent transcriptome analyses identified several hundred such genes [6-8]. The numbers of identified genes and their expression patterns varied among studies depending on experimental conditions and statistical criteria used. In our study [8] we noted that the expression of most quorum-controlled genes increased in the stationary phase of growth and could not be advanced to logarithmic phase by the addition of exogenous signal, confirming earlier observations with individual quorum-controlled genes [9-11]. This suggested that the activation of most quorum-controlled genes is not triggered by the accumulation of signal, and seems to require additional factors.

**Figure 1:**
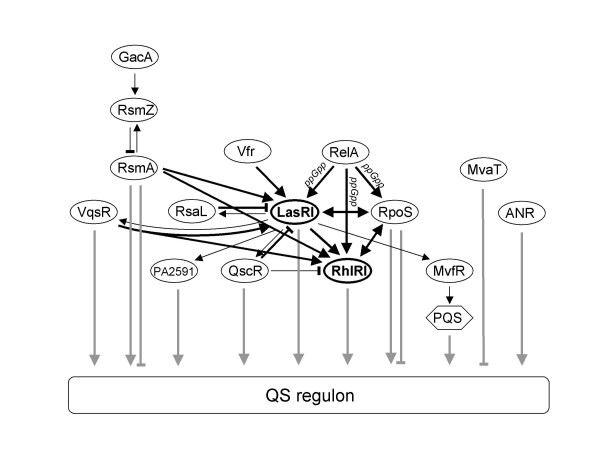
**The relationship between QS and other regulatory pathways**. The QS regulon is defined as the collection of genes activated by 3OC12-HSL-LasR or C4-HSL-RhlR, directly or indirectly. The different regulatory pathways shown affect the expression of overlapping subsets within the QS regulon. Effects on signal synthase and receptor expression have not been detailed separately. The bold black lines indicate superregulation of LasR-LasI and RhlR-RhlI. The bold grey lines indicate co-regulation of target genes. PA2591 is one of several predicted transcriptional regulators activated by *las *QS. This figure has been modified from [18].

In accordance with this hypothesis, several other regulatory systems such as the GacAS-RsmAZ two-component signal transduction pathway [12-14], the stationary phase sigma factor RpoS [9,15], and MvfR (PqsR)-quinolone signaling [16,17], have been shown to influence quorum-controlled gene expression in *P. aeruginosa*. They are thought to affect *las *or *rhl *mediated QS at two distinct levels of signal integration, either at the level of the regulators LasR and RhlR themselves, or at the level of complex QS target promoters [18] (Fig. 1). The stationary phase sigma factor RpoS, for example, appears to affect QS gene expression at both levels [15]. Furthermore, a recent study showed that there is an inhibitory substance (or substances) in complex medium which delays the expression of many quorum-controlled genes [19], but it is not clear at which level this occurs. Because transcripts of *lasR*, *rhlR*, and *rpoS *increase at the onset of stationary phase in concert with activation of most quorum-controlled genes [8,15], it is possible that the abundance of the encoded proteins is critical for target gene induction. On the other hand, we predict that those late genes for which these regulators are not limiting early in growth are co-regulated by other factors at the target promoter level. To better understand the network structure of the QS regulon, we expressed LasR, RhlR, and RpoS from a heterologous promoter in logarithmic phase and compared quorum-controlled gene expression in the corresponding strains to that of the wildtype by using DNA microarrays.

## Results and discussion

### LasR, RhIR and RpoS levels increase in the stationary phase of growth

**Figure 2:**
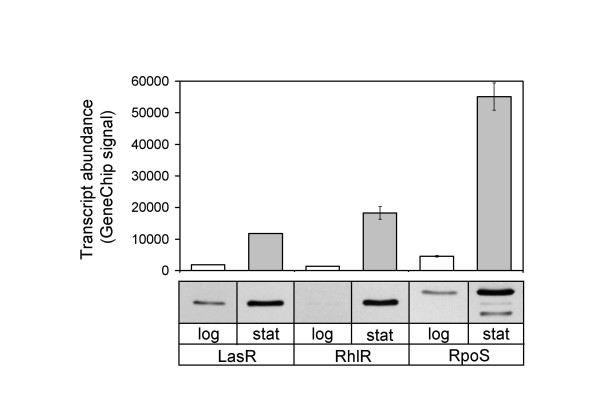
**Transcript and protein levels of LasR, RhlR, and RpoS during growth of *P. aeruginosa***. Top panel, *Pseudomonas *GeneChip signal levels; bottom panel, immunoblots of clarified lysates with LasR, RhlR, and RpoS specific antibodies. The two low molecular weight bands detected by RpoS antibody in stationary phase lysates represent partially degraded RpoS.

Our previous transcriptome analyses [8,15], in addition to other studies with reporter fusions [20-22], showed that the transcript levels of *lasR*, *rhlR*, and *rpoS *increase at the transition from logarithmic to stationary phase during batch culture growth of *P. aeruginosa*. Immunoblotting with LasR, RhlR, and RpoS-specific antisera showed that this expression pattern correlates well with the respective protein levels (Fig. 2).

### Expression strategy

If LasR, RhlR or RpoS levels were limiting the expression of QS-controlled genes during logarithmic growth of wildtype *P. aeruginosa*, early expression of these factors should advance quorum-controlled gene expression from stationary to logarithmic phase. To test this hypothesis, we constructed strains for regulatable expression of LasR, RhlR, and RpoS. Each allele, under the control of an arabinose-inducible *araBAD *promoter, was inserted in single copy into a neutral site of the chromosome of a *lasR*, *rhlR *mutant or an *rpoS *mutant, respectively. This arrangement allowed the careful titration of expression levels in logarithmic phase such that they matched those of the wildtype in stationary phase. For LasR and RhlR, it also allowed us to assess the impact of each regulator on gene expression independently and uncoupled from the QS hierarchy, where LasR-LasI is required for RhlR-RhlI expression [20,23,24]. We have thoroughly validated this approach by monitoring target gene expression and regulator protein levels in the engineered strains and in the parent strain throughout growth (see below).

### Many QS genes can be advanced by early expression of LasR, RhIR, or RpoS, but not by signal addition

**Figure 3:**
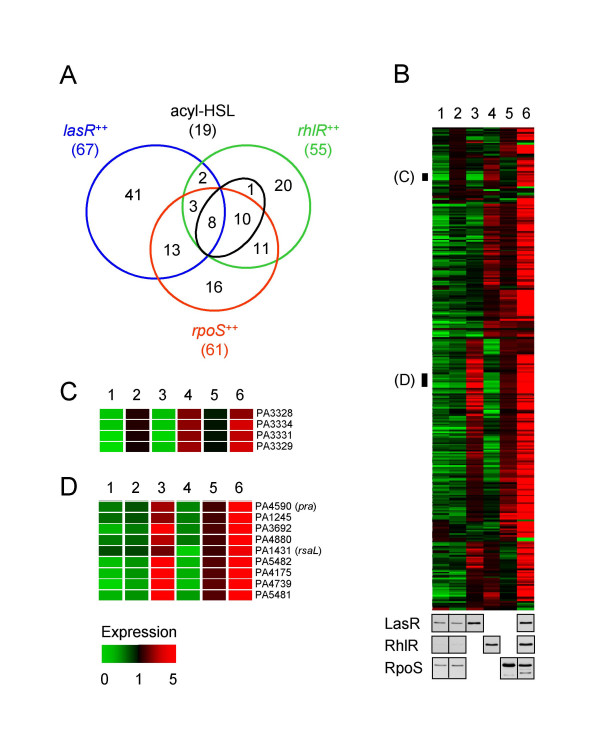
**Advancement of quorum-controlled gene expression**. A. Venn diagram showing the numbers of genes significantly induced by acyl-HSL addition or by early expression of LasR, RhlR, or RpoS. Comparison was relative to the PAO1 parent strain without exogenous signal in logarithmic phase. B. Top panel, *P. aeruginosa *transcript profiles (absolute GeneChip signal levels) of all previously identified 315 quorum-activated genes [8] under various growth and expression conditions. Strains were incubated in the presence of arabinose with or without 2 μM 3OC12-HSL and 10 μM C4-HSL. Lane 1, PAO1 parent without signals in logarithmic phase (50 mM arabinose); lane 2, PAO1 parent with signals in logarithmic phase (50 mM arabinose); lane 3, *lasR*, *rhlR *mutant expressing *lasR *with signals in logarithmic phase (7.5 mM arabinose); lane 4, *lasR*, *rhlR *mutant expressing *rhlR *with signals in logarithmic phase (50 mM arabinose); lane 5, *rpoS *mutant expressing *rpoS *with signals in logarithmic phase (25 mM arabinose); lane 6, wildtype without signals in stationary phase (1 mM arabinose). Bottom panel, immunoblots showing the corresponding protein levels of LasR, RhlR and RpoS under the conditions described above. C and D, Transcript profiles of individual genes enlarged from B.

Early expression of LasR and RhlR each resulted in the premature induction of 125 and 127 genes, respectively, during growth of *P. aeruginosa *in culture (p < 0.001 using Cyber-T analysis). Sixty-seven of the genes induced by LasR and 55 of the genes induced by RhlR are among the set of 315 quorum-induced genes we identified in a previous study [8] (Fig. 3A and Additional file 1). Early expression of RpoS resulted in the premature induction of 124 genes. Sixty-one of these genes were identified as quorum-controlled, and 36 are among the genes previously identified as RpoS-induced [15]. Thus, early expression of any of the three proteins revealed genes that are not among the previously characterized QS and RpoS regulons. Although this finding warrants further investigation, for the purpose of this study we focused on the set of 315 previously identified and well characterized quorum-regulated genes [8].

A heat map shows that the early expression of LasR, RhlR, and RpoS induced many genes in logarithmic phase to levels close to those of the wildtype in stationary phase (Fig. 3B). Nineteen genes were also activated by the addition of 3OC12-HSL and C4-HSL signals alone, but their induction levels were comparatively low and all of them could be induced further by early expression of RhlR (Fig. 3). Early expression of RpoS often only partially activated those genes that were more highly activated by LasR or RhlR. Because RpoS also has a small effect on LasR and RhlR expression [15], it appears that RpoS is capable of advancing many genes only indirectly through LasR and RhlR. Overall, there was a good correlation between the genes induced by early expression of LasR or RhlR, and their signal specificity determined by addition of 3OC12-HSL or both 3OC12-HSL and C4-HSL to a signal generation mutant [8]. For example, a cluster of *rhl*-specific genes was advanced by C4-HSL-RhlR but not by 3OC12-HSL-LasR (Fig. 3C), whereas several *las*-specific genes were advanced by 3OC12-HSL-LasR but not C4-HSL-RhlR (Fig. 3D). However, there were exceptions. PA5481 and PA5482, for example, can be advanced by 3OC12-HSL-LasR but not C4-HSL-RhlR (Fig. 3D) although in a signal synthesis mutant they responded better to both signals than to 3OC12-HSL alone [8]. Such genes may require the binding of both regulators to their promoters for activation, or they may require additional factors that are themselves under the control of 3OC12-HSL-LasR.

Among the genes significantly induced by early expression of LasR, RhlR, or RpoS were 47% of all genes activated early by addition of signals to cells in conditioned medium [19]. The large majority of this subset was induced by early expression of RhlR. RhlR was one of the genes triggered by media conditioning [19] and it also induced in minimal medium [24]. Thus, the advancement of QS in conditioned medium appears to be partially mediated through super-regulation of RhlR.

### Many other QS genes cannot be advanced significantly by early expression of LasR or RhIR

Most quorum-controlled genes showed little to no activation by LasR, RhlR or RpoS expression in logarithmic phase. One-hundred and twenty of these genes are late genes. Their quorum-dependent (and in most cases RpoS-independent) induction in the wildtype strain is delayed until stationary phase (Additional file 2). These late genes could not be advanced although several are predicted to be directly activated by LasR or RhlR as they possess conserved sequence elements, so-called *las-rhl *boxes, in their promoter regions [7,8,11]. Thus it appears that 3OC12-HSL-LasR, C4-HSL-RhlR, and RpoS levels are not limiting for the activation of many quorum-controlled genes.

**Figure 4:**
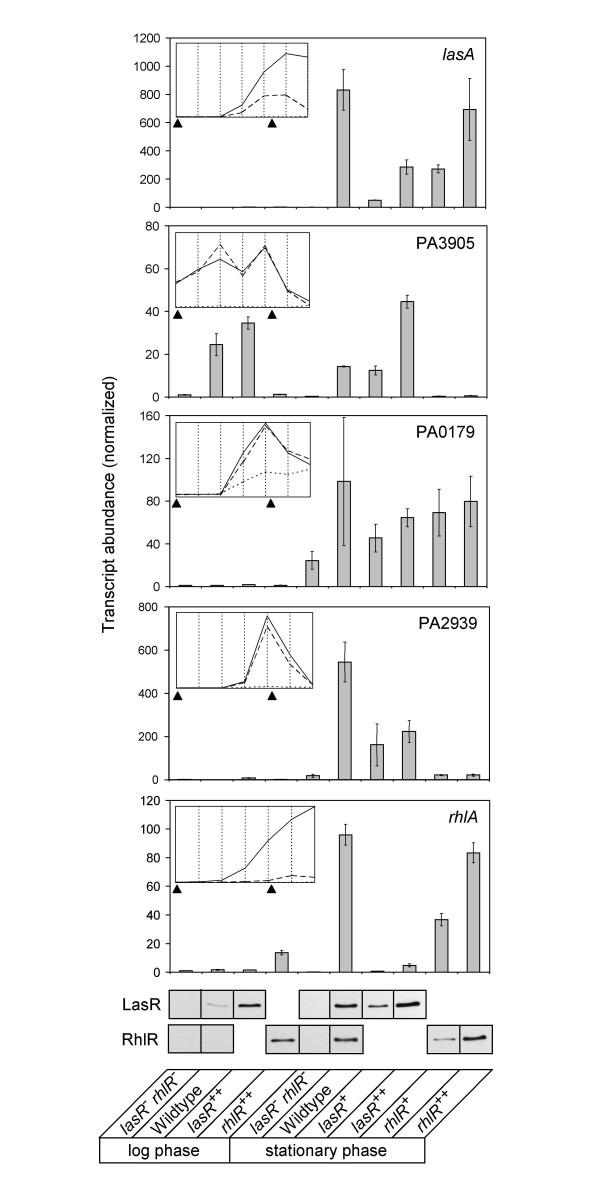
**Effect of acyl-HSL addition or early expression of LasR or RhlR on the transcription of selected *P. aeruginosa *quorum-controlled genes in logarithmic and stationary phase**. Top panels, transcript abundances as determined by real-time PCR normalized to the transcript levels of the *lasR, rhlR *mutant in logarithmic phase. Error bars indicate standard errors of the mean. Concentrations of arabinose added to the cultures were as follows (from left to right, in mM): 50, 50, 7.5, 50, 1.0, 1.0, 0.1, 0.4, 0.4, 1.6. For comparison, the insets in each panel show signal specificities previously determined in a *lasI, rhlI *signal generation mutant without exogenous signal (dotted line), with 2 μM 3OC12-HSL (dashed line), or with both 2 μM 3OC12-HSL and 10 μM C4-HSL (solid line) [8]. The black arrows indicate positions on the growth curve at which the corresponding cultures for real-time PCR analysis were harvested (left arrow, OD_600 _= 0.2; right arrow, OD_600 _= 2). Bottom panel, immunoblots showing the respective protein levels of LasR and RhlR.

To demonstrate that late quorum-controlled genes can still be activated in stationary phase using our engineered expression strains, and to confirm our microarray results, we measured the logarithmic and stationary phase transcript profiles of several quorum-controlled genes by real-time PCR. The genes *lasA*, PA0179, PA2939, and *rhlA*, which showed little to no quorum activation in logarithmic phase, could be activated in stationary phase cultures of strains expressing *lasR *or *rhlR *from the pBAD promoter (Fig. 4). Both *lasA *and *rhlA *possess a *las-rhl *box [7,8,25]. PA3905 is an example of one of the few early genes that are induced fully in the wildtype strain in logarithmic phase. Ectopic expression of *lasR *resulted in a similar level of induction in stationary phase.

As indicated above, there was generally a good correlation between regulator specificity and acyl-HSL signal specificity (as determined by early expression of LasR or RhlR in a signal receptor mutant and by addition of 3OC12-HSL or both signals to a signal synthesis mutant, respectively). An exception is PA0179, which was 3OC12-HSL specific [8] but responded to both LasR and RhlR expression in logarithmic phase (Fig. 4). This suggests that RhlR-C4-HSL is capable of activating PA0179, but this effect is masked in the signal generation mutant because 3OC12-HSL-LasR is required for RhlR expression and 3OC12-HSL-LasR already saturates the PA0179 promoter. Hence, our expression strategy provides a different assessment of QS promoter specificity because it functions independently of the QS cascade.

### Direct versus indirect regulation

We hypothesized that most genes advanced by QS are directly activated by QS. If this hypothesis is true, then we should be able to identify conserved sequence elements, so-called *las-rhl *boxes, upstream of these genes. In our previous study, we identified *las-rhl *box like sequences in 40 out of the 168 predicted quorum-controlled promoters [8]. Many of these promoters are associated with QS advanced genes. Of the 53 predicted promoters advanced by LasR, 16 contain a *las-rhl *box-like sequence, and of the 26 predicted promoters advanced by RhlR, 17 contain a *las-rhl *box-like sequence. Thus, it appears that genes advanced by RhlR are for the most part also directly regulated by this transcription factor, whereas genes advanced by LasR are mostly indirectly regulated (i.e. genes directly regulated by LasR are more evenly distributed among the genes that can and cannot be advanced). However, our data do not provide evidence for indirect regulation by LasR via a regulatory cascade. Candidate regulatory genes *mvfR*, PA2591, and *vqsR*, which are under direct transcriptional control by LasR [7,17,26,27], are not among the genes activated early (Additional file 1).

**Figure 5:**
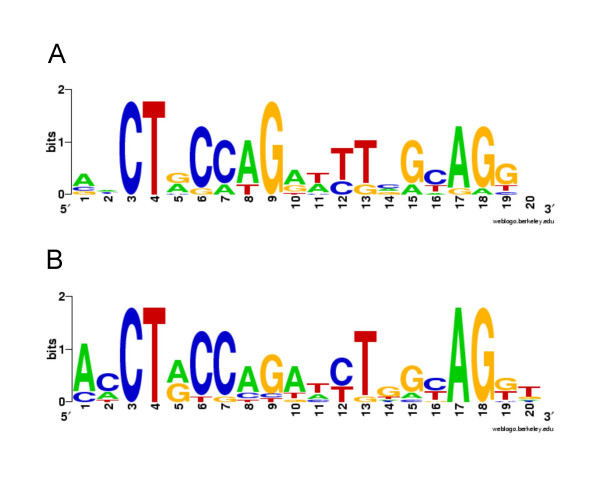
**Graphical representation of sequence alignments**. (A) RhlR consensus based on the weight matrix of sequences identified upstream of promoters most highly induced by RhlR early in growth. (B) *las-rhl *box consensus from our previous study [8]. The *las-rhl *box alignment is based on 11 previously identified sequences presumed to bind either LasR, RhlR or both. The overall height of the stack indicates sequence conservation, while the height of symbols within the stack indicates the relative frequency of each nucleic acid.

Our previous search of *las-rhl *box like sequences did not take into account differences between *las*-specific and *rhl*-specific binding sites. It is therefore possible that we missed several actual LasR or RhlR binding sites. Our identification of a subset of genes advanced by either LasR or RhlR in this study afforded the opportunity for a *de-novo*, unbiased search for separate LasR and RhlR consensus sequences. We selected those genes most highly induced by LasR alone or by RhlR alone and subjected the presumed upstream regulatory sequences to the pattern discovery algorithm CONSENSUS [28]. A sequence pattern that resembles known binding sites was identified for RhlR-advanced but not for LasR-advanced promoters (Fig. 5). This result confirms the notion that many genes advanced by RhlR are activated directly. Most genes advanced by LasR may be activated indirectly as mentioned above, or LasR binding sites may be more complex. Our recent binding studies with purified LasR support the latter. We noticed a general lack of sequence conservation and identified two subpopulations of *las*-specific binding sequences based on their distinct interactions with LasR [29]. Such heterogeneity undoubtedly complicates the identification of a single consensus sequence. Further biochemical characterization of a larger number of binding sites will be required for a more elaborate sequence alignment. Our alignment of RhlR sequences shows the conserved CT(N_12_)AG motif recognized previously [7,8,11,30], and implies several other conserved nucleotides (Fig. 5). Similarity to the original *las-rhl *box sequence alignment is high, reflecting a bias towards *rhl*-responsive genes in the original alignment [8].

In a second step, the matrix generated with CONSENSUS was used to search the upstream regions of all previously identified quorum-controlled genes. Fourty-two predicted QS promoters showed a significant match. Not surprisingly, most sequences had also been identified in our previous study [8]. In addition, we identified sequences with similarity to the RhlR consensus upstream of the following genes: PA2081, PA2146, PA3361, PA4141, PA4217 (qsc132), and PA5356 (*glcC*). Of these, the latter three were found to be advanced by RhlR.

## Conclusion

When *P. aeruginosa *is grown in complex medium, LasR, RhlR and RpoS protein levels increase in stationary phase (Fig. 2), correlating with the activation of most quorum-controlled genes. Our approach to investigating the advancement of quorum sensing gene expression was to adjust the expression levels of regulators in log-phase cells to match the levels in stationary phase. This is an overexpression strategy in which proteins are maintained within physiological levels known to exist in *P. aeruginosa*. Many QS genes could be activated in logarithmic phase by the early expression of LasR, RhlR, and RpoS, but not by signal addition. Thus, the levels of these three proteins can be critical in modulating the quorum-response in *P. aeruginosa*. Several of the genes were induced by more than one regulator, confirming overlapping specificities and co-regulation. Genes that directly and exclusively respond to QS are likely among the subset of genes whose maximal expression can be advanced by increased expression of LasR or RhlR during logarithmic phase. In fact, our results suggested that most genes advanced by RhlR are directly regulated by this transcription factor, allowing us to build a RhlR consensus sequence.

Many other QS genes fail to be advanced by 3OC12-HSL-LasR and C4-HSL-RhlR, although it has been shown that some of these genes are directly regulated by these signal and response systems. This confirms and extends a recent observation for an individual gene, *rhlA*, which is not significantly activated in logarithmic phase even when C4-HSL-RhlR is present [31]. These results suggest that the corresponding promoters are co-regulated by other transcription factors, likely constituting a network motif known as a multi-input dense overlapping regulon [32]. Some of the regulatory inputs may affect translation rather than transcription, as is the case for small regulatory RNAs that modulate quorum sensing gene expression [33,34]. The overall topology allows for specific responses to a multitude of signals, and may provide the basis for the exceptional environmental adaptability of *P. aeruginosa*. It also provides a simple explanation for the seemingly discordant sets of quorum-controlled genes identified by two separate groups under different culture conditions [7,8,35].

Taken together, our results indicate that co-regulation of target genes is an important, and perhaps predominant, feature of the *P. aeruginosa *QS network in addition to the well established super-regulation of the central components, LasR-LasI and RhlR-RhlI. Thus, a thorough understanding of the QS network will necessitate a comprehensive analysis of target promoter architecture, which should include the global identification of transcription factor binding sites. Technologies such as ChIP-chip, chromatin immunoprecipitation and microarray analysis [36], make this approach feasible.

## Methods

### Bacterial strains, plasmids, and culture conditions

Bacterial strains and plasmids are shown in Table 1. For plasmid and strain constructions, bacteria were grown in Luria-Bertani broth (LB). Where appropriate, antibiotics were added to maintain plasmids and to select for recombination or integration events. For transcript profiling experiments, *P. aeruginosa *strains were grown in 250 ml flasks containing 50 ml LB buffered with 50 mM 3-(N-morpholino) propanesulphonic acid, pH 7.0, at 37°C with vigorous shaking. Inocula were from mid-logarithmic phase cultures. The initial optical densities (OD_600_) were 0.02. Synthetic 3OC12-HSL and C4-HSL (2 and 10 μM final concentrations, respectively) and L-arabinose (0.4 to 50 mM final concentration) were added to cultures at the time of inoculation as indicated. Different concentrations of arabinose were necessary to induce each individual promoter to the appropriate level.

**Table 1:** Bacterial strains and plasmids

Strain or plasmid	Relevant property	Reference or origin
*Escherichia coli*		
DH5α	F^-^, φ80d*lacZ*ΔM15 Δ(*lacZYA-argF*)U169	Invitrogen
	*deoR recA*1 *endA*1 *hsdR*17(rk^-^, mk^+^)	
	*phoA supE*44 λ^- ^*thi*-1 *gyrA*96 *relA*1	
S17-1	*recA pro hsdR *RP4-2-Tc::Mu-Km::Tn7	[45]
*Pseudomonas aeruginosa*		
PAO1	Wild type	[46]
PAO1Δ*lasR*	PAO1 derivative; Δ*lasR*::Tc^R^	[47]
PAO1Δ*rhlR*	PAO1 derivative; Δ*rhlR*::Gm^R^	[47]
PAO1Δ*rhlR*Δ*lasR*		PAO1 derivative; Δ*rhlR*::Gm^R^ Δ*lasR*::Tc^R^
[47]		
PAO-*rpoS*	PAO1 derivative; *rpoS*::Gm^R^	[15]
MSC100	PAO1 derivative; Δ*rhlR*Δ*lasR*, p*BADlasR*	This study
MSC110	PAO1 derivative; Δ*rhlR*Δ*lasR*, p*BADrhlR*	This study
MSC120	PAO1 derivative; *rpoS*::Gm^R^, p*BADrpoS*	This study
Plasmids		
pFLP2	Source of Flp recombinase; Ap^R^	[37]
pJN105	*araC*-p*BAD *cloned in pBBR1MCS-5; Gm^R^	[41]
pJN105.*lasR*	*lasR *in pJN105	[48]
pJN105.*rhlR*	*rhlR *in pJN105	This study
pJN105.*rpoS*	*rpoS *in pJN105	This study
pSW196	mini-CTX1 based plasmid containing	[40]
		*araC*-p*BAD *from pBAD30; Tc^R^
pSW196.*lasR*	*lasR *in pSW196	This study
pSW196.*rhlR*	*rhlR *in pSW196	This study
pSW196.*rpoS*	*rpoS *in pSW196	This study

Strains for regulatable expression of LasR, RhlR, and RpoS were constructed as follows: Alleles of *lasR*, *rhlR*, and *rpoS *were placed under control of an arabinose-inducible promoter and inserted in single copy into the chromosome of *P. aeruginosa *using a specialized integration-proficient plasmid system [37,38]. The strains contained mutations in the respective chromosomal loci; i.e. *lasR *or *rhlR *were expressed in PAO *lasR rhlR*, and *rpoS *was expressed in PAO *rpoS*. Because of constraints with antibiotic resistance markers, *lasR *and *rhlR *expression constructs were first introduced into an isogenic PAO *rhlR *single mutant. To construct a double mutant background, a chromosomal *lasR *mutation was introduced into these strains by transformation with chromosomal DNA isolated from a PAO *lasR *mutant.

The *lasR *ORF was amplified from *P. aeruginosa *PAO1 genomic DNA by polymerase chain reaction (PCR) using primers 5'-N_6_GAATTCTGA*TTAACTTTA*TA*AGGAGG*AAAACATATG GCCTTGGTTGACGGTTTTC-3' and 5'-N_6_GCGGCCGCGGCAAGATCAGAGAGTAATAA GAC-3'. The underlined sequences indicate EcoRI and NotI restriction sites, respectively. The sequences in italics indicate a T7 gene enhancer element and an optimized ribosomal binding site (RBS) [39]. These sequences were included to enhance translation of LasR because expression levels from the native RBS (as assessed by Western blotting) were low even when induced fully. The *rhlR *and *rpoS *alleles were subcloned from pJN105.*rhlR *and pJN105.*rpoS*. These plasmids were constructed previously (see below). The *lasR *PCR product was cut with EcoRI and NotI, and pJN105.*rhlR *and pJN105.*rpoS *were cut with EcoRI-NotI and KpnI-SpeI, respectively. The digested fragments containing *lasR*, *rhlR*, or *rpoS *were ligated with appropriately cut pSW196 [40], which contains an arabinose-inducible *araBAD *promoter inserted into plasmid mini-CTX1 [38]. The resulting plasmids pSW196.*lasR *and pSW196.*rhlR *were each mobilized into PAO *rhlR*, and pSW196.*rpoS *was mobilized into PAO *rpoS *via *E. coli *S17-1. Mini-CTX1 encodes a site-specific integrase which mediates insertion at the chromosomal *attB *site, and it also contains Flp recombinase target sites flanking the multiple cloning site thus allowing *in vivo *excision of unwanted plasmid backbone DNA. To accomplish excision, pFLP2, encoding Flp recombinase, was introduced into the *P. aeruginosa *strains containing the respective *lasR*, *rhlR*, or *rpoS *expression constructs. This plasmid was then cured via the pFLP2-encoded *sacB *counterselectable marker. To determine whether strains were constructed properly, the size of fragments encompassing the *attB *region was determined by PCR with the primers 5'-CGTACAACGTGCCGGATATCG-3' and 5'GCTTCGGGATAAGCCAATCCTG-3' and subsequent agarose gel electrophoresis. In contrast to *lasR *and *rhlR *constructs, the *rpoS *expression construct did not insert at the *attB *site, even after repeated attempts. It did insert, however, at another, unknown site. We were unable to determine the exact insertion site by arbitrary PCR. We verified that the transcript profile of the *rpoS *mutant carrying the *rpoS *construct under non-inducing conditions was very similar to that of the original *rpoS *mutant, indicating that the insertion did not result in significant unintended gene expression changes. Transcription of neighboring chromosomal DNA from the *araBAD *promoter is minimized by strong T4 transcriptional terminators [38]. To generate PAO *lasR rhlR *double mutant backgrounds, a *lasR*::Tc^R ^mutation was introduced by transformation of chemically competent cells with 80 μg of genomic DNA isolated from PAO *lasR*::TcR as described [15].

The vectors pJN105.*rhlR *and pJN105.*rpoS *were constructed as follows: The *rhlR *and *rpoS *ORFs including native ribosomal binding sites were amplified from PAO1 genomic DNA by PCR. The primers for *rhlR *were 5'-N_6_GAATTCATCGATCAGGGCTTACTGCAATG-3' and 5'-N_6_TCTAGAGCGCTTCAGATGAGACCCAGC-3' with the underlined sequences indicating EcoRI and XbaI restriction sites, respectively. The primers for *rpoS *were 5'-N_6_GCTAGCAAGGGATAACGACATGGCAC-3' and 5'-N_6_GAATTCTCACTGGAACAGCGC GTCACT-3' with the underlined sequences indicating NheI and EcoRI restriction sites, respectively. The resulting PCR fragments were cut with the indicated restriction enzymes and inserted into the multiple cloning site of appropriately digested plasmid pJN105 [41] to give pJN105.*rhlR *and pJN105.*rpoS*.

### Microarray experiments

Cultures were harvested at early logarithmic phase (OD_600 _= 0.2) and early stationary phase (OD_600 _= 2). RNA isolation as well as subsequent cDNA synthesis, labeling, and *P. aeruginosa *GeneChip genome array (Affymetrix, Santa Clara, CA) processing were performed as described previously [8,15]. Each experiment was done in duplicate. A control experiment confirmed that the addition of arabinose had no significant effect on gene expression. The GeneChip profiles of *P. aeruginosa *in logarithmic phase with and without arabinose showed the same correlation as those of two replicate cultures grown in the absence of arabinose (not shown). Data were processed with the Affymetrix GeneChip Operating Software 1.1. The web-based program Cyber-T [42] was used for statistical analysis as described [15]. The p-value threshold was 0.001, and the corresponding confidence estimate termed posterior probability of differential expression, PPDE (<p), was ≤ 0.97. This p-value was chosen based on graphical analysis of the distribution of p-values and their association with the non-uniform distribution (indicating differential expression) and the uniform distribution (indicating no differential expression) [42]. To determine which quorum-controlled genes qualify as late genes, we demanded that transcript levels in stationary phase were significantly higher in wildtype *P. aeruginosa *compared to a *lasR, rhlR *mutant, and that this difference was larger in stationary phase than in logarithmic phase. Global gene expression patterns ("heat-maps") were constructed with GeneSpring 7.2 (Agilent Technologies, Palo Alto, CA). Transcript profiles of individual genes were normalized to the median expression level. Microarray data were deposited in the EMBL ArrayExpress repository (Accession number E-MEXP-1183).

### Real-time PCR

*P. aeruginosa *culture conditions, as well as RNA isolation and cDNA synthesis procedures were identical to those used for microarray analysis. Real-time PCR was performed with an ABI Prism 7900 Sequence Detection System (Applied Biosystems, Foster City, CA). Cycling parameters were 10 min at 95°C followed by 40 cycles of 15 s at 95°C and 1 min at 60°C. Dissociation profiles of the amplified products were run to evaluate non-specific amplification. Each PCR reaction contained 1× SYBR Green Master Mix (Applied Biosystems), 1 ng cDNA template, and 7.5 pmol of each primer in a 25-μl volume. Gene-specific primers were designed and data were analyzed using Primer Express and SDS 2.1 software, respectively (Applied Biosystems). Relative transcript levels were determined by using the standard curve method and by using the *nadB *transcript as a calibrator. Standard curves were constructed with 10^-4 ^to 10 pg of RNA-free genomic DNA purified from *P. aeruginosa *PAO1 (Genomic-tip kit, Qiagen). Experiments were performed in duplicate.

### Immunoblotting

For protein analysis, samples were withdrawn from the same *P. aeruginosa *cultures that were used for transcript profiling. Cells were harvested by centrifugation. Pellets were suspended in lysis buffer containing 25 mM Tris-HCl, pH 7.8, 150 mM NaCl, 1 mM EDTA, 1 mM dithiothreitol, 0.5% Tween-20, 10% glycerol, 2 μM 3OC12-HSL and 10 μM C4-HSL. The suspensions were sonicated, and the resulting lysates subjected to ultracentrifugation at 100,000 × g for 15 min. Protein concentrations were determined by using the Bradford assay. Approximately 2 μg of each supernatant fraction was separated by 12.5% SDS-PAGE. The separated proteins were blotted onto a nylon membrane. The membrane was treated with polyclonal antibodies against LasR, RhlR, or RpoS. Proteins were detected by using a secondary anti-rabbit horseradish peroxidase-conjugated IgG and chemiluminescent substrate (Pierce, Rockford, IL). Antibodies against *P. aeruginosa *LasR and RhlR were generated in rabbits immunized with His_10_-LasR or His_10_-RhlR affinity-purified under denaturing conditions. Polyclonal rabbit antibody against *P. aeruginosa *RpoS was obtained from V. Venturi [43].

### Consensus sequence analysis

Ten predicted promoter regions (-400 to -1 relative to the translational start) associated with the genes most highly induced by either LasR alone or by RhlR alone (Additional file 1) were each subjected to the pattern search algorithm CONSENSUS [28]. Overlap of sequences with upstream open reading frames was disallowed. A matrix length of 16 bp was chosen for the final alignment, because it yielded the most consistent results. The highest scoring matrix from the final cycle was used to depict a sequence alignment in WEBLOGO [44]. The same matrix was also used in PATSER [28] to identify sequences in upstream regulatory regions of other quorum-controlled genes. The default weight score of 7 was used as the lower threshold.

## Authors' contributions

MS designed the study, carried out experiments and drafted the manuscript. EPG conceived of the study, participated in its design and coordination and helped to draft the manuscript. All authors read and approved the final manuscript.

## Supplementary Material

Additional file 1 Table S1. Quorum-controlled genes that can be advanced by signal addition or LasR, RhlR or RpoS overexpression. This table lists *P. aeruginosa *quorum-controlled genes that are expressed early in growth when acyl-HSL signals are added or when LasR, RhlR, or RpoS are expressed ectopically. Click here for file

Additional file 2 Table S2. Late quorum-controlled genes that cannot be advanced by signal addition or LasR, RhlR, or RpoS overexpression. This table lists *P. aeruginosa *quorum-controlled genes that are not expressed early in growth even when acyl-HSL signals are added or when LasR, RhlR, or RpoS are expressed ectopically. Click here for file
